# Identification and characterization of phages parasitic on bradyrhizobia nodulating groundnut (*Arachis hypogaea* L.) in South Africa

**DOI:** 10.1016/j.apsoil.2016.09.010

**Published:** 2016-12

**Authors:** Levini A. Msimbira, Sanjay K. Jaiswal, Felix D. Dakora

**Affiliations:** a Department of Crop Sciences, South Africa; b Department of Chemistry, Tshwane University of Technology, Pretoria, South Africa

**Keywords:** Biotic factor, Rhizobiophage, Siphoviridae, Phylogeny, *Bradyrhizobium*, *nif*H and *gln*II genes

## Abstract

In this study, three lytic phages (namely, PRSA-1, PRSA-2 and PRSA-26) were isolated and characterized for their morphology, host range, profile and restriction endonuclease banding pattern of genome size. The susceptible rhizobial isolates were identified by *nif*H and *gln*II sequence analysis. The results showed that all phages had polyhedral head with non-contractile tail which confirmed their relationship with the *Siphoviridae* family. All the three phages produced highly distinct plaques on their host bradyrhizobial lawn, and were highly sensitive to chloroform. The phage genome sizes ranged from 34.7 to 53.1 kbp. The phages were tested against groundnut-nodulating bradyrhizobial strains TUTAHSA75, TUTAHSA155 and TUTAHSA126 isolated from South African soils. The results revealed different bacterial susceptibilities to phages. Bradyrhizobial isolate TUTAHSA126 was susceptible to all three phages (i.e. PRSA-1, PRSA-2 and PRSA-26), TUTAHSA155 to two phages (i.e. PRSA-1, PRSA-2), and TUTAHSA75 to only one phage (i.e. PRSA-1). Phylogenetic analysis of *nif*H and *gln*II gene sequences of the phage-susceptible bradyrhizobial isolates revealed their close relatedness to a diverse group of *Bradyrhizobium* species. Phage PRSA-1 could parasitize on all three bradyrhizobial strains, which indicates its potential role in horizontal gene transfer through lysogenic conversion, and/or genetic transduction in soil microbial environments.

## Highlights

• Rhizobiophages affect symbiotic relationship of rhizobia and legumes.
• It is the first report of lytic phages parasitized on groundnut nodulating rhizobia.
• All phages confirmed their relation with the *Siphoviridae* family.
• Phage genome sizes were varied from 34.7 to 53.1 kbp.
• The susceptible rhizobial isolates were identified as diverse group of *Bradyrhizobium*.

## 1 Introduction

The legume/rhizobia symbiosis is important for sustainable agriculture, especially its N contribution in cropping systems. So far, most studies have focused on abiotic factors affecting the symbiosis. As a result, very little information currently exists on biotic constraints affecting the microsymbiont (Wielbo et al., 2012). *Rhizobium*-lysing viruses (or rhizobiophages) constitute the most important biotic factor affecting rhizobial numbers and legume nodulation in agricultural soils (Shahaby et al., 2014). It has been estimated that there are about 10^7^–10^9^ viruses g^−1^ soil, which by comparison is 5–1000-fold higher than bacteria present in soils (Williamson et al., 2013). Given this large population of rhizobiophages in soil, symbiotic N contribution can be significantly altered in cropping systems if they parasitize on root-nodule bacteria in legume rhizosphere. Additionally, these viruses can also lyse bacterial cells, and alter their genetic diversity through transduction and/or lysogenic conversion (Williamson et al., 2005).

Bacteria are susceptible to infection by a group of phages called bacteriophages. But root-nodule bacteria can exhibit different levels of susceptibility to these phages or viruses (Hashem and Angle, 1988b). Bacteriophages that infect rhizobia (termed rhizobiophages) were first reported by Gerretsen et al. (1923) and have subsequently been isolated for all the major groups of root-nodule bacteria (Staniewski, 1987). Rhizobiophages have been implicated in the control of rhizobial populations in soil (Allen and Allen, 1950); so through their lytic activity, they can decrease N_2_ fixation in legumes.

Rhizobiophages can vary significantly in their morphology, host range, and general characteristics (Staniewski, 1987). With about 5500 phage isolates of known morphology, rhizobiophages constitute the largest of all virus groups (Ackermann and Prangishvili, 2012). Symbiotically defective mutants of rhizobia were isolated as surviving cells after exposure to specific virulent phages, and although morphological and physiological changes occurred, the mechanism underlying loss of symbiotic properties are still unknown (Barnet, 1979, Raleigh and Signer, 1982).

Phages associated with susceptible rhizobial strains were first isolated from rhizosphere soil by Schmidt et al. (1986) and have since been used to characterize indigenous rhizobial populations (Appunu and Dhar, 2006, Jaiswal et al., 2012). The occurrence and distribution of phages active against various groups of rhizobia have been reported in India (Dhar et al., 1980, Dhar et al., 1993, Singh et al., 1980, Jaiswal and Dhar, 2010), Poland (Staniewski, 1970), New Zealand (Patel, 1976), Canada (Lesley, 1982) and Senegal (Lajudie and Bogusz, 1984). Recently, phages infective on common bean rhizobia have also been isolated and characterized in Mexico (Santamaría et al., 2014). Since the discovery of phages that infect rhizobia, no study has reported on phages preying on groundnut rhizobia. Therefore, the aim of this study was to isolate and identify phages infecting indigenous groundnut-nodulating bradyrhizobia in South African soils.

## 2 Materials and methods

### 2.1 Isolation of bradyrhizobia and rhizobiophages

Groundnut (*Arachis hypogea* L.) nodules were collected from Klipladrift in Mpumlanga Province of South Africa, and rhizobia isolated from those nodules as described by Somasegaran and Hoben (1994). The nodulation ability of each isolate was confirmed by plant-infection test using pot (containing sterilized sand) experiment under glasshouse conditions with 14 h light and 10 h darkness (Vincent, 1970). The Groundnut seeds were surface-sterilized with 95% ethanol for 10 s and 3% NaOCl for 2 min followed by rinsing with sterilized water several times. The sterilized seeds were transferred into potted sand. After germination of seeds, seedling was inoculated with 1 ml (10^7^ cells per ml) of rhizobial culture. Un-inoculated pots were considered as controls. Plants were watered with Broughton and Dilworth (1971) N-free medium. Plants were harvested after 5 weeks to see the nodulation. Rhizobia were re-isolated from nodules of plants used for authentication and maintained on YMA slant containing 0.3% CaCO_3_ for further study. The soil from which phages and bradyrhizobia were isolated had pH 4.85, and contained 224 mg/kg Ca, 50 mg/kg Mg, 135 mg/kg K and 7 mg/kg Na.

Rhizobiophages were isolated from rhizosphere soils collected from the same experimental fields at Klipladrift by the single-plaque method of double agar layer (Dhar et al., 1978). The soil and nodule samples were crushed in 10 ml water using mortar and pestle and suspension filtered through cotton swabs to remove any debris. The filtered soil suspension was left to stand for 8–10 h to settle down any soil particles and the supernatant removed and centrifuged at 10,000*g* for 20 min to remove any soil debris. Following centrifugation, the supernatant was filtered using 0.22 μm pore size membranes (Sartorius Biolab, Germany), and 0.2 ml of this membrane filtrate mixed with 0.1 ml rhizobial culture in 3 ml melted YMA (45 °C), and over-layered on previously prepared YMA plates. The phages were purified by three successive isolations of single plaque.

### 2.2 Host range of rhizobiophages

Rhizobial strains isolated from root nodules of different legumes (including groundnut) and from different locations were examined for their host range using the isolated rhizobiophages. Petri dishes each containing a basal layer of YM agar were plated with various exponentially-growing rhizobial culture (0.1 ml) suspended in 3 ml melted YMA medium (45 °C) which contained 0.7% agar. Shortly after the agar solidified, 50 μl of phage suspension (ca. 2 × 10^7^ pfu/ml) was spotted on the overlay of YMA. All plates were incubated at 28 ± 2 °C for 5–6 days, and lytic zones in the spotted areas were examined for susceptible host strains.

### 2.3 Chloroform sensitivity

For chloroform sensitivity, 10 ml of filtered phage suspension were mixed with 1.0% chloroform (v/v), shaken vigorously for 1 min, and the solution kept at 28 ± 2 °C for 24 h. The suspension was then centrifuged and supernatant was filtered through 0.22 μm pore size membrane filter. The filtered solution was mixed with 0.1 ml rhizobial culture in 3 ml melted YMA (45 °C), and over-layered on previously prepared YMA plates to know surviving phage particles (pfu/ml).

### 2.4 Electron microscopy

The morphology of the isolated phages was studied using transmission electron microscopy (TEM). High titer (10^8^–10^9^ pfu/ml) of filtered phage lysate of each isolate was used for TEM studies. High titer phages were prepared by the confluent lysis method (Jaiswal and Dhar, 2010). A 25 μl of each prepared phage sample was placed on 200-mesh carbon-coated copper grids and allowed to absorb for 5 min. The phage samples were negatively stained with 5% aqueous uranyl-acetate for 5 min, examined, and photographed with a JEOL transmission electron microscope (TEM, JEM 3100F at 200KV). The size of phage virion was calculated as the mean of five measurements (n = 5).

### 2.5 Isolation of phage genome

A freshly prepared 1 ml high titer (10^8^–10^9^ pfu/ml) filtered phage solution was used to isolate phage genomic DNA. A confluent lysed plate was flooded with 5 ml phage buffer (10 mM Tris–HCl, 10 mM MgSO_4_, 68 mM NaCl and 1 mM CaCl_2_), and kept at 4 °C for 4 h. The phage buffer (containing phage) was carefully removed from the plates while avoiding any pieces of agar, and poured into Eppendorf tubes. The Eppendorf tubes were centrifuged at 5000*g* using a microcentrifuge (Eppendorf centrifuge 5424R). The supernatant was filtered with nylon membrane filter paper (0.22 μm porosity, Sartorious, Germany), and the filtrate used to extract phage DNA by using DNA clean and Concentrator kit (Zymo research, USA).

### 2.6 Restriction digestion of phage DNA

Fast digest restriction endonucleases obtained from Thermo Scientific (Lithuania) were used to digest phage DNA as recommended by the manufacturer. About 500 ng of DNA was added to 50 μl reaction mixture and the suspension was subjected to electrophoresis in a 0.9% agarose gel stained with ethydium bromide. The electrophoresis was done in 1× TAE (Tris-acetic acid EDTA) buffer at 5 V/cm. Lambda DNA/EcoRI + HindIII marker 3 of Thermo Scientific (Lithuania) was used as a molecular weight marker. The size of phage genome was estimated by summing up all the restriction-digested fragment lengths (bp) using Gel imager software (BioRad, USA).

### 2.7 Isolation of rhizobial DNA and PCR amplification of *nif*H and *gln*II region

Bacterial genomic DNA was extracted using GenEluet bacterial DNA isolation kit (Sigma Aldrich, USA) according to the manufacturer’s instructions. Polymerase chain reaction (PCR) was carried out with 40–60 ng DNA in 25 μl reaction volume containing 5 μl (5×) My Taq PCR buffer, 0.1 μl Taq polymerase (5U) (Bioline, USA), 1 μl (10 pM) of each primer, and sterilized double-distilled water with Thermal cycler (T100, Bio-Rad USA). The details of primers and temperature profiles are indicated in Table 1.The amplified products were estimated on horizontal gel electrophoresis of 1.5% agarose gel stained with 1 μg ml^−1^ ethidium bromide with standard DNA marker (GeneDirex, 100 bp ladder) and photographed using gel documentation system (Geldoc™ XR+, Bio-RAD, USA).

### 2.8 Sequencing of *nif*H and *gln*II genes and their phylogenetic analysis

The PCR-amplified products of *nif*H and *gln*II genes were purified using Favour/Prep PCR purification kit (FAVORGEN, Sigma USA). The purified samples were sequenced (Macrogen, Netherlands), and the quality of all sequences checked using BioEdit 7.0.0 software (Hall, 2004). The NCBI GenBank databases were used to identify closely related species to the test samples by means of using BLAST_n_ program. The sequences were deposited in the NCBI GenBank database to get accession numbers. The nucleotide sequences of *Bradyrhizobium*-type strains were selected to align with sample sequences to enable us construct phylogenic trees using MEGA 6.0 programe (Tamura et al., 2013). Those phylogenetic trees were generated by the Kimura-2 parameter model (Kimura, 1980) using the Maximum-Likelihood methodalgorithm with 1000 bootstraps (Felsenstein, 1985).

## 3 Results and discussion

In this study, we showed that rhizobiophages in South African soils can parasitize on N_2_-fixing bradyrhizobial cells with an ability to reduce their numbers and thus affect nodulation and N_2_ fixation. To our knowledge, this is the first study to describe the presence and activity of phages in South African soils, and is also the first report of phages infective on groundnut-nodulating bradyrhizobia. The presence of phages capable of parasitizing and reducing the number of N_2_-fixing bradyrhizobia in South African soils has implications for lowering N_2_ fixation and N contribution in this legume in traditional cropping systems (Allen and Allen, 1950, Hashem and Angle, 1988a).

**Fig. 1:**
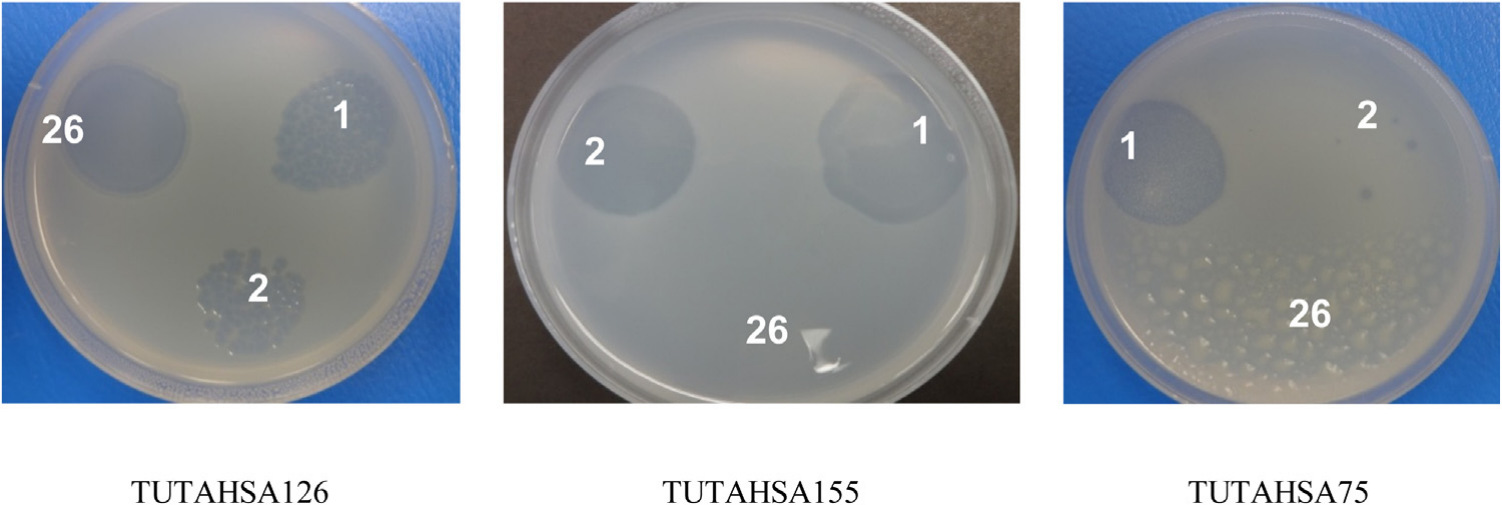
Cross infectivity of phages across the groundnut rhizobial strains: 1 = PRSA-1; 2 = PRSA-2; 26 = PRSA-26. The numbers indicate the phage lysed plaque on the plate.

From soil suspension bioassays, some drops formed plaques or hollow zones on the bacterial lawn of YMA plates. Out of the 47 bacterial isolates tested, only three (namely TUTAHSA75, TUTAHSA126 and TUTAHSA155) formed hollow zones with soil suspension, and therefore exhibited susceptibility to phage invasion. The isolation and purification of the viruses resulted in three distinct strains of phages, which were specific in their infectivity of the bradyrhizobial host. Designated as phages PRSA-1, PRSA-2 and PRSA-26, strain PRSA-1 was found to be parasitic on all three bradyrhizobial isolates (TUTAHSA155, TUTAHSA75 and TUTAHSA126), while phage PRSA-2 lysed isolates TUTAHSA155 and TUTAHSA126, and PRSA-26 was strictly infective on only bradyrhizobial strain TUTAHSA126 (see Fig. 1). However, phage PRSA-1 showed strong lytic activity with bradyrhizobial isolates TUTAHSA155 and TUTAHSA75, while PRSA-2 and PRSA-26 were highly lytic with only TUTAHSA155 and TUTAHSA126, respectively, in yeast mannitol broth.

All the three phage strains produced distinct plaques with their respective hosts. Phage PRSA-1 characteristically formed the largest plaque (2 mm diameter) on bradyrhizobial strain TUTAHSA155, while PRSA-26 produced the smallest plaque (0.5 mm diameter) with TUTAHSA126 (Table 2). Furthermore, none of the 70 root-nodule bacteria isolated from soybean (30), groundnut (20) and common bean (20) in South Africa and Ethiopia showed susceptibility to the three phages (PRSA-1, PRSA-2 and PRSA-26), which indicates their potential for use as inoculants where soils may be infested with rhizobiophages. The exhibition of a very narrow host range for the phages indicates a strict and highly lytic activity on only the bacterial symbionts from the original homologous groundnut host, where the phages were isolated. This finding is consistent with earlier reports by Barnet (1972), Patel (1976), Dhar and Ramkrishna (1987), and Santamaría et al. (2014), which showed restriction in phage activity on rhizobial isolates.

All the three phage isolates were highly sensitive (100%) to chloroform, with no plaques formed after treating each phage solution with 1% (v/v) chloroform. This was in contrast to the untreated phage solutions, which produced plaques with their respective bradyrhizobial hosts. The high sensitivity to chloroform probably suggests the absence of lipids in the phage particles (Kęsik-Szeloch et al., 2013), and is consistent with the report by Ackermann (2006) which found that one-third of tailed phages were chloroform-sensitive.

**Fig. 2:**
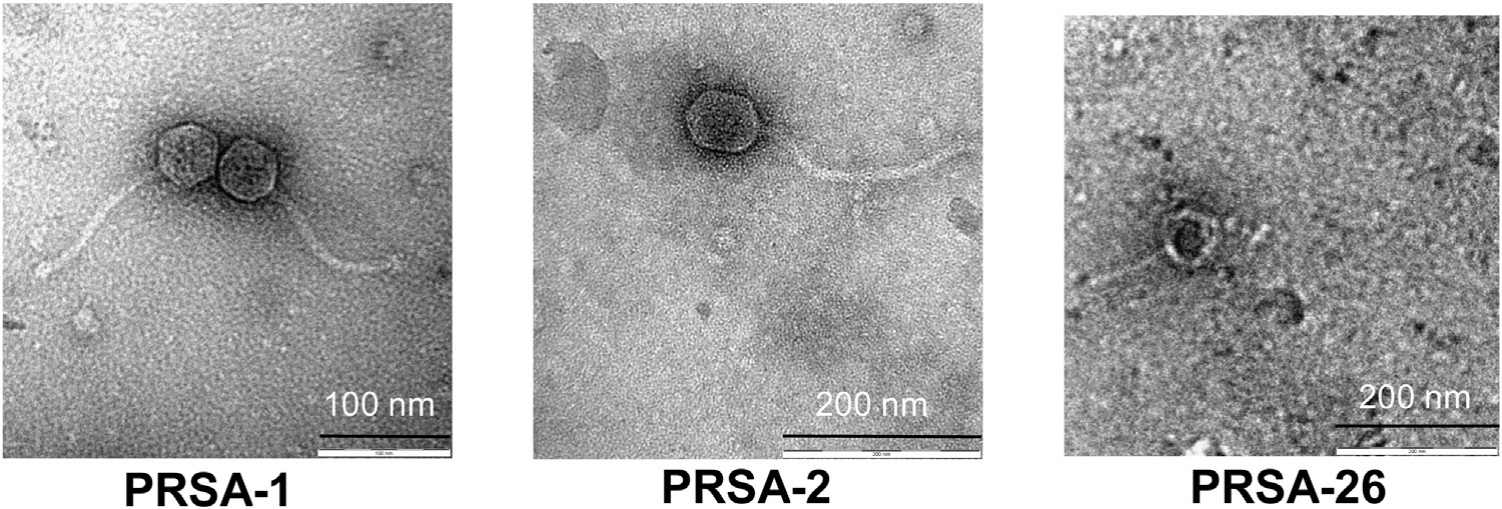
Electron micrographs of negatively stained isolated rhizobiophages.

TEM micrographs of the three phages (PRSA-1, PRSA-2 and PRSA-26) revealed polyhedral heads with flexible non-contractile tails of differing sizes (Fig. 2), which clearly placed them in the Siphoviridae group (Ackermann, 2006). Phages PRSA-1 and PRSA-2 characteristically had tailfins, while PRSA-26 had none (Table 2). Notwithstanding the slight differences in head and tail dimensions, the TEM data revealed close similarity of these phage isolates to phage SR-2, which was reported to parasitize on *Bradyrhizobium,* and phage 2011 which parasitized on *Rhizobium melilotii* (Werquin et al., 1988, Appunu and Dhar, 2008).

**Fig. 3:**
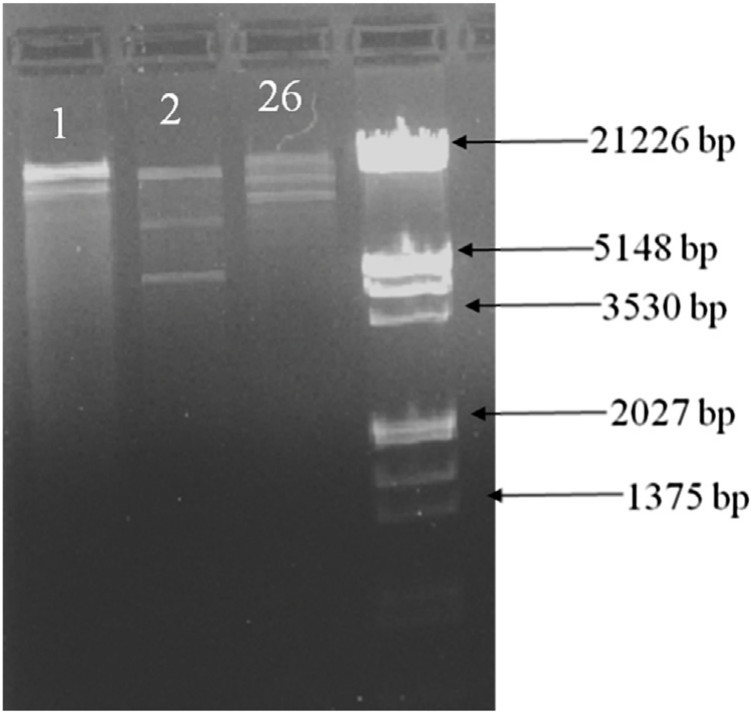
Restriction endonuclease *EcoR*I + *Hind*III digestion pattern of phage genome 1 = PRSA-1; 2 = PRSA-2 and 26 = PRSA-26.

Except for double digestion with EcoRI *+* HindIII, none of the 16 restriction enzymes used (namely, GsuI, BsuRI, BfoI, AluI, BamHI, HpaII, HinfI, HhaI, MspI, HaeIII, RsaII, HaeII, TaqI, BpmI, EcoRI and HindIII) could digest genomic DNA from the phages. These results suggest that the genomes of the test phage isolates probably had no restriction sites for the fourteen other endonucleases used in this study. It is likely that the genome of these phages carried DNA modifications (including methylation) that probably made them resistant to these restriction enzymes (Jaiswal and Dhar, 2010, Kęsik-Szeloch et al., 2013, Santamaría et al., 2014). But more importantly, the unrelated restriction banding patterns obtained in this study could also suggest that all the three phages differed significantly in their nature and profile. When the double-digested DNA was visualized on agarose gel, a total of eight bands were observed (Fig. 3), which were all polymorphic in nature. The sum of fragment sizes appearing in a gel were used to estimate the molecular weight of each phage DNA, and these were found to vary from ∼34.7 kbp in PRSA-2 to ∼53.1 kbp in PRSA-26 (Table 2). The double-stranded DNA from restriction endonuclease digestion of the phage genome with EcoRI + HindIII is a common feature of tailed phages (Jaiswal and Dhar, 2010, Santamaría et al., 2014).

**Fig. 4:**
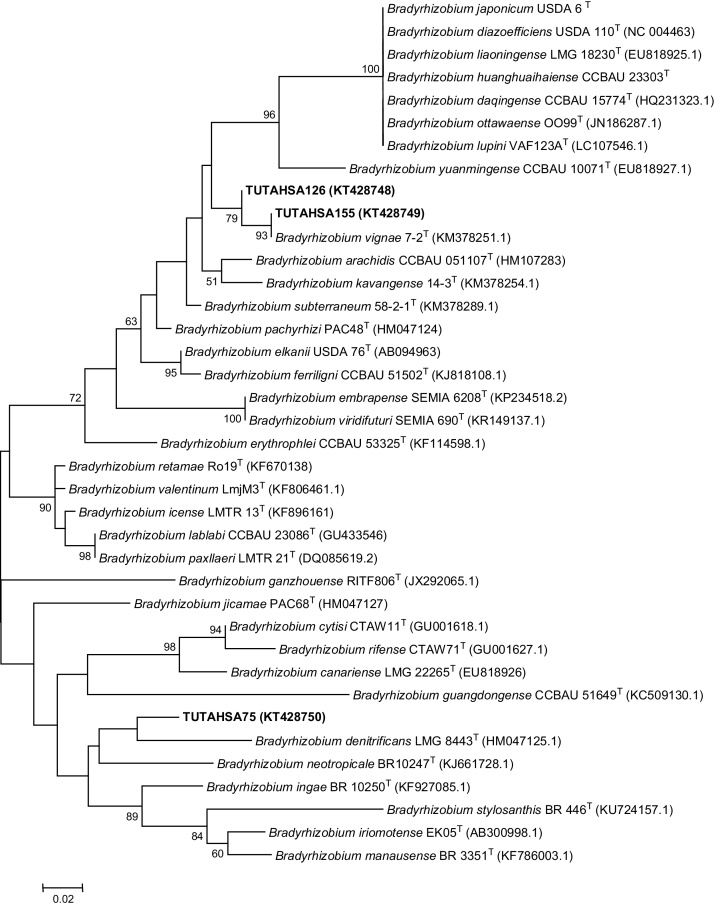
Maximum-Likelihood phylogeny for phage susceptible groundnut-nodulating bradyrhizobia based on *nifH* nucleotide sequence data.

**Fig. 5:**
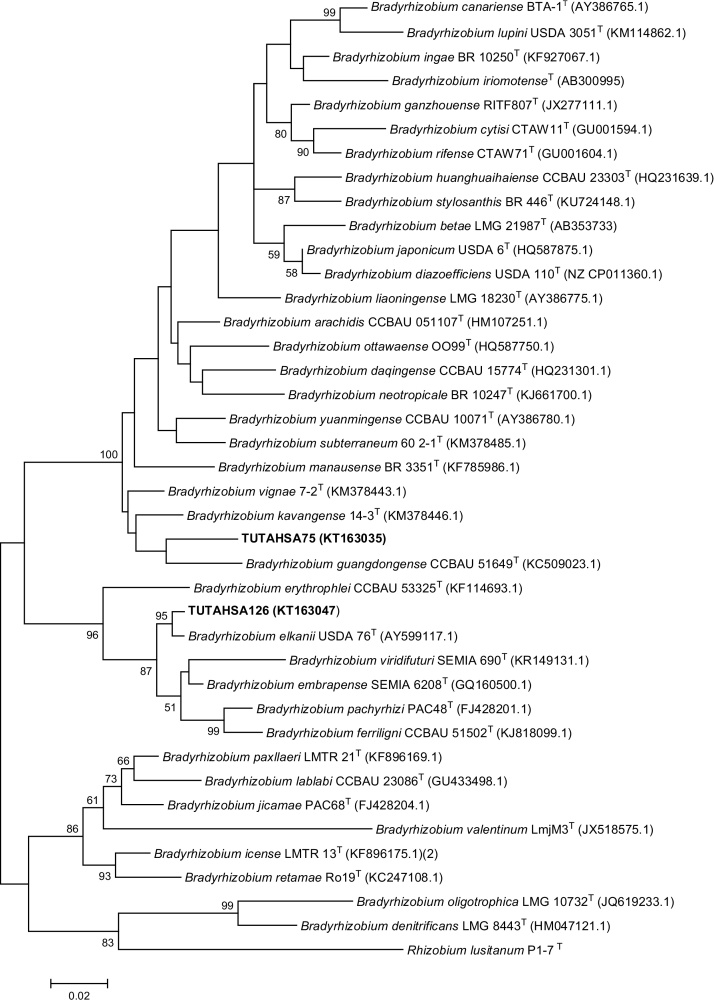
Maximum-Likelihood phylogeny for phage susceptible groundnut-nodulating bradyrhizobia based on *gln*II nucleotide sequence data.

PCR-amplified products of the three phage-susceptible bradyrhizobial isolates (namely TUTAHSA75, TUTAHSA126 and TUTAHSA155) yielded single bands of 880 bp and 680 bp for *nif*H and *gln*II genes respectively. However, the PCR amplification failed for the *gln*II region of strain TUTAHSA155. The sequences generated from analysis of *nif*H and *gln*II genes aligned with *Bradyrhizobium*–type sequences in the NCBI GenBank. The contents of T, C, A and G in the nucleotides of *nif*H gene recorded mean frequencies of 18.8, 27.1, 19.6 and 34.4%, respectively. Theses *nif*H sequences showed 59.6% conserved, 40.4% variable and 33.0% parsimony-informative region. The *gln*II gene similarly contained 19.4, 30.3, 17.1 and 33.2% mean frequencies of T, C, A and G nucleotides, respectively, and had 62.7% conserved, 37.32% variable and 28.72% parsimony-informative region. The phylogenetic tree constructed from *nif*H gene sequences using the neighbour-joining method showed that isolates TUTAHSA155 and TUTAHSA126 were very closely related, and grouped with *Bradyrhizobium vignae* with a high 93 bootstrap support, while strain TUTAHSA75 clustered with *Bradyrhizobium denitrificans* (Fig. 4). The *nif*H topology however differed considerably with *gln*II phylogram, as in the latter, isolate TUTAHSA126 was closely aligned to *B. elkanii* with 95 bootstrap value, while TUTAHSA75 stood with *Bradyrhizobium guangdongense* (Fig. 5). Taken together, the phylogenetic study of *gln*II and *nif*H genes of phage-susceptible groundnut-nodulating bradyrhizobia has revealed a huge diversity in microsymbiont population, a finding consistent with the results of Yang et al. (2005) and Steenkamp et al. (2008). The phylogenies from *gln*II and *nif*H gene sequences seem to suggest that strains TUTAHSA126, TUTAHSA155 and TUTAHSA75 have their own unique and independent evolutionary origin.

The ability of the phage isolates to differentiate between the test bacteria is indicative of the genetic variability among the *Bradyrhizobium* strains nodulating groundnut in South Africa. The isolation and wide testing of rhizobiophages for their ability to parasitize on diverse rhizobia has the potential to identify phage-resistant, symbiotically very effective strains for inoculant production. Although nodulation failure under field conditions is often attributed to a range of biotic and abiotic factors, including low rhizobial populations, never have rhizobiophaghes been included as a causal factor in poor nodulation of field legumes. The evidence from this study clearly shows that rhizobiophages exist in South African soils that can reduce nodulation and N_2_ fixation in field-grown groundnut.

Even if the phages have no immediate significant effect on the soil population of the non-homologous rhizobia, it is possible that, in the course of evolution, interactions with mutants of susceptible strains could lead to gene transfer, and thus result in susceptibility. A better understanding of rhizobiophage ecology can help to reduce the negative effects of phages on rhizobial symbiosis. Better still, the typing of rhizobial isolates against phages could be an easy way to characterize and identify phage-resistant strains since specificity is one of their basic characteristics, as shown in this study. In conclusion, this study is the first report on the presence and activity of rhizobiophages in South African soils, which parasitize on indigenous groundnut bradyrhizobia.

## Figures and Tables

**Table 1:** Primers and PCR temperature profile used in this study.

Primers	Sequences (5′–3′)	Target position	PCR temperature profiles	References
*nif*Hf	TACGGNAARGGSGGNATCGGCAA	*nif*H (28–51)	95 °C 5 min, 20X (94 °C 30 s, 65 °C decrease on 0.5 °C in each cycle 30s, 72 °C 1.5 min), 25X (94 °C 30 s, 55 °C 30s, 72 °C 1.5 min) 72 °C 10 min	Nzoué et al. (2009)
*nif*Hr	AGCATGTCYTCSAGYTCNTCCA	*nif*H(809–787)		
*gln*IIf	AAGCTCGAGTACATCTGGCTCGACG	*gln*II (13–38)	95 °C 2 min, 35X (95 °C 45 s, 65 °C 30s, 72 °C 1.5 min) 72 °C 10 min	Stępkowski et al. (2011)
*gln*IIr	SGAGCCGTTCCAGTCGGTGTCG	*gln*II (681–660)		

**Table 2:** Morphology and general characteristics of isolated phages against groundnut rhizobia.

	Phage strains		
	PRSA-1	PRSA-2	PRSA-26
Susceptible rhizobial strains	TUTAHSA75, TUTAHSA155, TUTAHSA126	TUTAHSA126, TUTAHSA155	TUTAHSA126
Plaque appearance	Round, clear and homogeneous	Round, clear and homogeneous	Round, clear and homogeneous
Plaque size (mm)	2	1	0.5
Phage morphology:			
Head	Hexagonal	Hexagonal	Oval
Tail	Long noncontractile	Long noncontractile	Long noncontractile
Phage dimensions			
Head diameter (nm)	58.51 ± 9.27	67.04 ± 11.56	77.27 ± 4.67
Tail length (nm)	131.16 ± 12.18	210.85 ± 20.92	126.45 ± 23.76
Tail width (nm)	9.04 ± 1.36	10.09 ± 3.79	9.79 ± 1.67
Chloroform sensitivity	Very sensitive	Very sensitive	Very sensitive
Genome size (kbp)	36.6	34.7	53.1

## References

[bib0005] Ackermann H.-W., Prangishvili D. (2012). Prokaryote viruses studied by electron microscopy. Arch. Virol..

[bib0010] Ackermann H.-W. (2006). Classification of bacteriophages. Bacteriophages.

[bib0015] Allen E.K., Allen O. (1950). Biochemical and symbiotic properties of the rhizobia. Bacteriol. Rev..

[bib0020] Appunu C., Dhar B. (2006). Differential symbiotic response of phage-typed strains of *Bradyrhizobium japonicum* with soybean cultivars. J. Microbiol. Seoul.

[bib0025] Appunu C., Dhar B. (2008). Morphology and general characteristics of lytic phages infective on strains of *Bradyrhizobium japonicum*. Curr. Microbiol..

[bib0030] Barnet Y.M. (1972). Bacteriophages of *Rhizobium trifolii* I: morphology and host range. J. Gen. Virol..

[bib0035] Barnet Y.M. (1979). Properties of *Rhizobium trifolii* isolates surviving exposure to specific bacteriophage. Can. J. Microbiol..

[bib0040] Broughton W., Dilworth M. (1971). Control of leghaemoglobin synthesis in snake beans. Biochem. J..

[bib0045] Dhar B., Ramkrishna K. (1987). Morphology and general characteristics of phages of chickpea rhizobia. Arch. Microbiol..

[bib0050] Dhar B., Singh B., Singh R., Srivastava J., Singh V., Singh R. (1978). Occurrence and distribution of rhizobiophages in Indian soils. Acta Microbiol. Pol..

[bib0055] Dhar B., Singh B., Singh R., Srivastava J., Singh R. (1980). Seasonal incidence of rhizobiophages in soils around Varanasi. Indian J. Exp. Biol..

[bib0060] Dhar B., Upadhyay K., Singh R.M. (1993). Isolation and characterization of bacteriophages specific for *Rhizobium leguminosarum* biovar *phaseoli*. Can. J. Microbiol..

[bib0065] Felsenstein J. (1985). Confidence limits on phylogenies: an approach using the bootstrap. Evolution.

[bib0070] Gerretsen F.C., Gryns A., Sack J., Sohn-Gren N.L. (1923). Das verkomrnen eines bakteriophagen in den wurzelknollehen der leguminosen. Zentralbl. Bakteriol. Parasitenkd. Infektionskr. Hyg. Abt..

[bib0075] Hall, T. (2004). BioEdit version 7.0. 0. Distributed by the author, website: www.mbio.ncsu.edu/BioEdit/bioedit.html.

[bib0080] Hashem F., Angle J. (1988). Rhizobiophage effects on *Bradyrhizobium japonicum*, nodulation and soybean growth. Soil Biol. Biochem..

[bib0085] Hashem F., Angle J. (1988). Rhizobiophage effects on *Bradyrhizobium japonicum*, nodulation and soybean growth. Soil Biol. Biochem..

[bib0090] Jaiswal S.K., Dhar B. (2010). Morphology and general characteristics of phages specific to *Lens culinaris* rhizobia. Biol. Fertil. Soils.

[bib0095] Jaiswal S.K., Anand A., Dhar B., Vaishampayan A. (2012). Genotypic characterization of phage-typed indigenous soybean bradyrhizobia and their host range symbiotic effectiveness. Microbial Ecol..

[bib0100] Kęsik-Szeloch A., Drulis-Kawa Z., Weber-Dąbrowska B., Kassner J., Majkowska-Skrobek G., Augustyniak D., Lusiak-Szelachowska M., Zaczek M., Górski A. (2013). Characterising the biology of novel lytic bacteriophages infecting multidrug resistant *Klebsiella pneumoniae*. Virol. J..

[bib0105] Kimura M. (1980). A simple method for estimating evolutionary rates of base substitutions through comparative studies of nucleotide sequences. J. Mol. Evol..

[bib0110] Lajudie P.d., Bogusz D. (1984). Isolation and characterization of two bacteriophages of a stem-nodulating *Rhizobium* strain from *Sesbania rostrata*. Can. J. Microbiol..

[bib0115] Lesley S. (1982). A bacteriophage typing system for *Rhizobium meliloti*. Can. J. Microbiol..

[bib0120] Nzoué A., Miché L., Klonowska A., Laguerre G., de Lajudie P., Moulin L. (2009). Multilocus sequence analysis of bradyrhizobia isolated from Aeschynomene species in Senegal. Syst. Appl. Microbiol..

[bib0125] Patel J. (1976). Morphology and host range of virulent phages of lotus rhizobia. Can. J. Microbiol..

[bib0130] Raleigh E.A., Signer E.R. (1982). Positive selection of nodulation-deficient *Rhizobium phaseoli*. J. Bacteriol..

[bib0135] Santamaría R.I., Bustos P., Sepúlveda-Robles O., Lozano L., Rodríguez C., Fernández J.L., Juárez S., Kameyama L., Guarneros G., Dávila G. (2014). Narrow-host-range bacteriophages that infect *Rhizobium etli* associate with distinct genomic types. Appl. Environ. Microbiol..

[bib0140] Schmidt E., Zidwick M.J., Abebe H. (1986). *Bradyrhizobium japonicum* serocluster 123 and diversity among member isolates. Appl. Environ. Microbiol..

[bib0145] Shahaby A.F., Alharthi A.A., El-Tarras A.E. (2014). Molecular characterization of rhizobiophages specific for *Rhizobium* sp. *Sinorhizobum* sp., and *Bradyrhizobium* sp. Int. J. Curr. Microbiol. Appl. Sci..

[bib0150] Singh R., Dhar B., Singh B. (1980). Morphology and general characteristics of viruses active against cowpea *Rhizobium* CB756 and 32H1. Arch. Virol..

[bib0155] Somasegaran P., Hoben H.J. (1994). Counting rhizobia by a plant infection method. Handbook for Rhizobia.

[bib0160] Stępkowski T., Żak M., Moulin L., Króliczak J., Golińska B., Narożna D., Safronova V.I., Mądrzak C.J. (2011). *Bradyrhizobium canariense* and *Bradyrhizobium japonicum* are the two dominant rhizobium species in root nodules of lupin and serradella plants growing in Europe. Syst. Appl. Microbiol..

[bib0165] Staniewski R. (1970). Typing of Rhizobium by phages. Can. J. Microbiol..

[bib0170] Staniewski R. (1987). Morphology and general characteristics of phages active against Rhizobium. J. Basic Microbiol..

[bib0175] Steenkamp E.T., Stepkowski T., Przymusiak A., Botha W.J., Law I.J. (2008). Cowpea and peanut in southern Africa are nodulated by diverse *Bradyrhizobium* strains harboring nodulation genes that belong to the large pantropical clade common in Africa. Mol. Phylogenet. Evol..

[bib0180] Tamura K., Stecher G., Peterson D., Filipski A., Kumar S. (2013). MEGA6: molecular evolutionary genetics analysis version 6.0. Mol. Biol. Evol..

[bib0185] Vincent, J.M. (1970). A manual for the practical study of the root-nodule bacteria. A manual for the practical study of the root-nodule bacteria.

[bib0190] Werquin M., Ackermann H.-W., Levesque R.C. (1988). A study of 33 bacteriophages of *Rhizobium meliloti*. Appl. Environ. Microbiol..

[bib0195] Wielbo J., Kidaj D., Koper P., Kubik-Komar A., Skorupska A. (2012). The effect of biotic and physical factors on the competitive ability of *Rhizobium leguminosarum*. Open Life Sci..

[bib0200] Williamson K.E., Radosevich M., Wommack K.E. (2005). Abundance and diversity of viruses in six Delaware soils. Appl. Environ. Microbiol..

[bib0205] Williamson K.E., Corzo K.A., Drissi C.L., Buckingham J.M., Thompson C.P., Helton R.R. (2013). Estimates of viral abundance in soils are strongly influenced by extraction and enumeration methods. Biol. Fertil. Soils.

[bib0210] Yang J.K., Xie F.L., Zou J., Zhou Q., Zhou J.C. (2005). Polyphasic characteristics of bradyrhizobia isolated from nodules of peanut (*Arachis hypogaea* L.) in China. Soil Biol. Biochem..

